# Antiviral Activity Exerted by Natural Products against Human Viruses

**DOI:** 10.3390/v13050828

**Published:** 2021-05-04

**Authors:** Maria Musarra-Pizzo, Rosamaria Pennisi, Ichrak Ben-Amor, Giuseppina Mandalari, Maria Teresa Sciortino

**Affiliations:** 1 Department of Chemical, Biological, Pharmaceutical and Environmental Sciences, University of Messina, Viale SS. Annunziata, 98168 Messina, Italy; mmusarrapizzo@unime.it (M.M.-P.); rpennisi@unime.it (R.P.); ichrak.benamor.etud@fss.usf.tn (I.B.-A.); 2 Shenzhen International Institute for Biomedical Research, 1301 Guanguang Rd. 3F Building 1-B, Silver Star Hi-Tech Park Longhua District, Shenzhen 518116, China; 3 Unit of Biotechnology and Pathologies, Higher Institute of Biotechnology of Sfax, University of Sfax, Sfax 3029, Tunisia

**Keywords:** viral infections, natural bioactive compounds, novel antiviral drugs, drug resistance

## Abstract

Viral infections are responsible for several chronic and acute diseases in both humans and animals. Despite the incredible progress in human medicine, several viral diseases, such as acquired immunodeficiency syndrome, respiratory syndromes, and hepatitis, are still associated with high morbidity and mortality rates in humans. Natural products from plants or other organisms are a rich source of structurally novel chemical compounds including antivirals. Indeed, in traditional medicine, many pathological conditions have been treated using plant-derived medicines. Thus, the identification of novel alternative antiviral agents is of critical importance. In this review, we summarize novel phytochemicals with antiviral activity against human viruses and their potential application in treating or preventing viral disease.

## 1. Introduction

A growing number of studies have shown the beneficial effects of plant-derived small organic compounds, named secondary metabolites. Most plant-derived compounds possess great potential in therapeutic applications for chronic diseases, degenerative processes, carcinogenesis, and antiviral activity [1]. A wide variety of active phytochemicals, including coumarins, flavonoids, terpenoids, organosulfur compounds, lignans, polyphenols, saponins, proteins, and peptides have been found to influence cellular functions, membrane permeability, and viral replication. Thus, naturally based pharmacotherapy may be a proper alternative for treating viral diseases [2,3,4]. Anti-viral drugs can be classified according to their chemical nature or their activity against viral proteins or cellular host proteins. In particular, the anti-viral activity can be exerted based on their capability to inhibit viral entry, viral DNA and RNA synthesis, as well as viral reproduction. The differences in the viral structure and replication cycle are crucial for the design of any antiviral drugs [5]. Enveloped viruses possess lipid-bilayer membranes and enter their host cells by fusion between the plasma membrane and the viral envelope. On the other hand, non-enveloped viruses enter the cytosol by endocytic mechanisms or directly penetrating the plasma membrane. The presence or absence of a viral envelope is likely to play a significant role in the effectiveness (or lack thereof) of the virucidal, since the exterior membranous-surface of the virus is what makes the first contact with the antiviral [6]. Moreover, host-targeting natural antivirals, which modulate cellular biological functions, can offer a broad-spectrum antiviral activity, reduced resistance phenomena but higher likelihood of toxicity [7]. The antiviral activity of a wide range of secondary metabolites and phytochemicals can be established through a variety of biological assays, commonly used to test the cytotoxicity, cytopathic effect, and the capability to block the cells to cells viral spread, thus limiting and/or fighting viral dissemination [8]. Purified natural products are considered a rich resource for novel antiviral drug development [9]. However, the extraction and isolation of a natural compound can be a difficult process, as many compounds are present in low concentration in the natural source. Besides, due to the complexity of the chemical structure, it is not easy to synthesize a natural compound. Furthermore, although natural compounds are mostly used as raw unpurified extracts, the isolation of biomolecules is of great importance for the prediction of their properties related to the absorption, distribution, metabolism, excretion, and toxicity of a drug molecule [10]. In the following sections, the antiviral activities of several natural products towards DNA and RNA viruses will be discussed with an analytical approach concerning the cellular/viral targets of the molecules and their impact on the infection and viral replication cycle.

## 2. Therapeutic Natural Compounds against DNA Viruses

### 2.1. Hepatitis B Virus (HBV)

Hepatitis B virus (HBV) is an enveloped virus from the *Hepadnaviridae* family with a 3.2 kb double-stranded DNA (dsDNA) genome. HBV is known to infect the liver and cause acute and chronic inflammation in hepatocytes. Chronic HBV-infection may lead to liver diseases, such as hepatocellular carcinoma (HCC) and cirrhosis. The HBV genome encodes the hepatitis B surface antigen (HBsAg), the hepatitis B envelope antigen (HBeAg), the hepatitis B core antigen (HBcAg), a viral polymerase harboring reverse transcriptase and DNA-dependent DNA polymerase activities, and the HBx protein. The viral nucleocapsid is made of many copies of the core HBcAg antigen and it is surrounded by a lipid envelope comprising of HBeAg and HBsAg. The first step in the HBV replication cycle is the attachment of the viral S1 receptors to heparin sulfate proteoglycans on liver cells, after which virions can penetrate the cells and the nucleocapsids are released into the cytoplasm and reach the nucleus where the circular DNA is released [11]. The DNA is then converted into pregenomic RNA and new viral proteins are synthesized. The new viral particles are released from host cells [12]. Currently, the therapy for HBV includes antiviral nucleoside or nucleotide analogues, such as lamivudine and entecavir as well as immunomodulators such as standard or PEGylated interferon-alpha (IFN-α) [13]. Although various therapies have been implemented, there are severe side effects, elevated costs, and a high drug resistance rate associated with current anti-HBV treatment [14]. Over the past few decades, natural products have been studied to identify new anti-HBV drugs (Table 1).

#### 2.1.1. Natural Products Inhibiting Viral Entry, Replication and Maturation of HBV Particles

The inhibitors of viral entry and fusion are receiving increasing attention for HBV due to its highly selective tropism. Methanolic extracts of *Hybanthus enneaspermus* inhibited HBs Ag binding [15]. Consistently, methanolic extracts from seeds of *Terminalia bellerica* and leaves of *Enicostemma axillare* have been shown to block the HBV DNA polymerase [15]. *Phyllanthus amarus* extracts were found to downregulate hepatitis B virus mRNA transcription and suppress hepatitis B virus polymerase activity and the release of the virus into Hep-G/2.2.15 cells [16]. It has been suggested that treatment with iminosugars interferes with the glycosylation of envelope proteins and reduces the infectivity of hepatitis viral particles [17]. Particularly, Jacob and colleagues demonstrated that a natural iminosugars 1-deoxynojirimycin (1-DNJ) derived from a silkworm (*Bombyx mori* L.) extract inhibited HBV particles maturation in a dose- and time-dependent manner [17]. Interestingly, the selectivity index (SI) of the silkworm extract was greater than for ribavirin, suggesting that the silkworm extract can be used as monotherapy or in combination with IFN-α for the treatment of HBV [17]. Recently, most researchers are focused on the development and screening of synthetic or naturally derived HBsAg secretion inhibitors. Indeed, the major obstacle in HBV clearance is represented by high rates of HBV surface antigen (HBsAg) secretion in chronic HBV infection. Ethyl acetate and chloroform fractions of *Boehmeria nivea* (BN) leaves extracts significantly inhibited HBeAg and HBsAg secretion into the medium and inhibited HBV DNA replication in Hep-G/2.2.15 cells with no cytotoxic effects [18].

#### 2.1.2. Natural Products Targeting Host Cellular Factors

Amongst others, betulinic acid is a good anti-HBV candidate. It has been shown that betulinic acid isolated from *Pulsatilla chinensis* enhanced ROS generation in the liver of transgenic mice by suppressing the manganese superoxide dismutase (SOD2) expression, with an inhibitory effect on HBV replication [19]. It has been found that *Curcuma longa L.* (CLL) extract inhibits the transcription of HBV X (HBx) gene through a p53-mediated pathway, with no cytotoxicity on liver cells. These results indicate CLL extract as an efficient herbal medicine against HBV [20]. The compound LPRP-Et isolated from *Liriope platyphylla* roots was observed to have potential anti-viral effect against HBV through a mechanism involving the NF-κB (nuclear factor kappa B) signaling pathway [21].

### 2.2. Herpes Simplex Virus (HSV-1)

Herpes simplex viruses have a worldwide distribution and are found in the most remote human populations. Amongst major viral infections, herpes simplex virus type 1 (HSV-1) continues to be a major public health problem, causing oral and genital infections associated with herpes labialis or cold sore. Indeed, the infection is common worldwide, with 45% to 98% of the world population being infected [22]. Viral particles are composed of double-stranded DNA and an icosahedral capsid, which is surrounded by an amorphous tegument and an envelope containing viral glycoproteins [22]. Due to the high prevalence of HSV-1 infections, several antiviral drugs have been developed for their treatment. It has been suggested that natural products from plants can exhibit anti-HSV-1 activities (Table 2). HSV-1, like a multitude of viruses, employ glycosaminoglycan (GAG) as initial attachment receptors during infection. Polyphenols are observed to target HSV-1 glycoprotein’s that interact with GAGs and, in turn, prevent their association with the cell surface GAGs as well as subsequent binding receptors [23]. Such natural products need to be isolated and screened for their potential to act as antiviral compounds [24].

#### 2.2.1. Natural Products Targeting Viral Gene Expression

An important natural antiviral agent against herpes is represented by curcumin, which can inhibit the HSV-1 replication [25,26]. In vitro studies of curcumin and its derivatives, namely gallium-curcumin and Cu-curcumin, exhibited remarkable antiviral activity against HSV-1 in cell cultures assays, decreasing the immediate-early (IE) gene expression and infectivity [26]. Curcumin has an effect on the recruitment of RNA polymerase II to IE gene promoters through the mediation of the viral transactivator protein VP16, by an independent process of p300/CBP histone acetyltransferase effect [26]. Extracts rich in polyphenols from raw shelled pistachios and the leaves of *Morus alba* and *Aloe vera* have a significant antiviral potential against HSV-1 [23,27,28]. It has been recently demonstrated that polyphenols from raw shelled pistachios (*Pistacia vera*. L.) interfere with the expression of HSV-1 viral proteins by preventing their transcription or translation, as well as with the viral DNA synthesis [27].

#### 2.2.2. Natural Products Blocking the Viral Entry

Flavonoids are widely distributed as secondary metabolites produced by plants. They are divided into several classes such as anthocyanidins, flavones, flavonols, flavanones, flavan, isoflavanoids, and abiflavanoids [29]. Among flavonols, the antiviral effect of quercetin is the most extensively investigated. Hung and colleagues have suggested possible mechanisms whereby quercetin may exert its anti-HSV activity [30]. They revealed that quercetin inhibits the infection of HSV-1, HSV-2 and acyclovir-resistant HSV-1 mainly by blocking viral binding and host cell penetration. Quercetin also suppresses NF-kB activation, which is essential for HSV gene expression. Similarly, the anti-binding effects of the almond skin extract from *Prunus dulcis* was demonstrated in Vero cells [31,32].

#### 2.2.3. Natural Products Targeting Host Cellular Factors

Homoharringtonine, isolated from *Cephalotaxaceae*, was reported to bind the phosphorylated serine 209 residues of the eukaryotic translation initiation factor-4E (eIF4E), thus inducing its degradation and inhibiting the first step of the elongation phase of eukaryotic translation [33]. Homoharringtonine possesses antiviral activity against HSV-1 with an IC_50_ value of 139 nM [34].

### 2.3. Human Papilloma Virus (HPV)

Human papillomaviruses (HPVs) are the most common sexually transmitted viral agents worldwide, infecting about 80% of the sexually active population. Specific types of high-risk human papillomaviruses, such as HPV type 16 and 18 (HPV16 and HPV18) are associated with cervical cancer [35]. HPVs are small, non-enveloped DNA viruses with a circular genome of around 8 kb, with eight overlapping open reading frames, comprising early (E), and late (L) genes and an untranslated long control region. The L1 and L2 genes encode the major and minor capsid proteins. The capsid contains 72 pentamers of L1 and approximately 12 molecules of L2 [36]. HPVs have a unique life cycle, which is tightly linked to the differentiation program of the infected host epithelium. HPV infection starts in the basal epithelium, basal cells, or stem cells in proliferation, where HPVs are in latent phase of infection and the viral genome is maintained at a low copy number without production of virions [37]. Polyphenols, from natural and herbal extracts, are raising great interest as a powerful and safe anticancer strategy towards cervical cancer [38] (Table 3).

#### 2.3.1. Natural Products with Direct anti-HPV Activity

The antitumor and antiviral properties of curcumin were assessed in HPV associated human cervical cancer cell lines. Curcumin showed an inhibitory activity against the expression of E6 and E7 oncogenes of HPV-16 and HPV-18 as two main highly oncogenic human papillomaviruses, resulting in loss of transforming phenotypes and reduction of cellular growth [39]. Two botanical formulations called "Basant”, containing purified curcumin, saponins mixed to *Emblica Officinalis*, *Aloe vera* extracts, *Mentha citrata* oil, “praneem", composed with purified saponins, extracts from *Azadirachta indica*, *Emblica Officinalis*, and *Aloe vera* mixed to *Mentha citrata* oil, were capable to block the transfer of HPV16 pseudovirions in HeLa cells [40]. The in vitro biological activities of *Ficus carica* fruit latex were explored onto cervical cancer CaSki and HeLa cell lines. Data showed that latex inhibits rapid growth and invasion and downregulates the expression of p16 and HPV oncoproteins E6, E7 [41].

#### 2.3.2. Natural Products Promoting Apoptosis and Inhibiting Cellular Transcriptional Factors

Green tea, one of the most widely consumed beverages, has been proven to possess beneficial anticarcinogenic and antiproliferative properties attributed to the biological properties of green tea polyphenolic compounds. Epigallocatechin-3-gallate (EGCG) is the major bioactive polyphenol present in green tea [42]. One of the first studies on the effect of EGCG on the HPV-16 associated cervical cancer cell line CaSki, demonstrated that the EGCG antiproliferative effect depends on the arrest of the cell cycle in the G1 phase, leading to programmed cell death by apoptosis [43]. The lipid-soluble rhizome extract of *Pinellia pedatisecta* promoted apoptosis by the increase of expression of caspase-8, caspase-3, Bax, p53 and p21 on the HPV16 and HPV18-positive CaSki and HeLa cell lines, respectively [44]. Other studies proved that *Cudrania tricuspidata* stem extract induces apoptosis and have a cytotoxic effect in HPV-16 positive SiHa cancer cells, with no cytotoxic effect on HaCaT human normal keratinocytes at concentrations of 0.125–0.5 mg/mL [45]. Additionally, curcumin inhibited IkBα phosphorylation and degradation, thus preventing the activation of NF-κB and leading to apoptosis. Moreover, curcumin downregulated the activator protein-1 (AP-1) binding activity in HeLa cells with a decreasing effect on the transcription of HPV-18 [46]. The AP-1 transcriptional factor regulates the epithelial tissue-specific gene expression of almost all HPV types. These data indicated that curcumin, through apoptosis modulation and the downregulation of viral oncogenes, can be a good candidate for the management of highly oncogenic HPV infections. Fractionated extracts of *Bryophyllum pinnata* leaves, crude extracts of *Phyllanthus emblica* fruits and *Kaempferia parviflora* ethanolic extracts had an anti-HPV activity by inhibition on AP-1 and the cellular proto-oncogenic transcription factor STAT3, which is crucial for the cell cycle progression and cervical carcinogenesis [47,48,49].

### 2.4. Adenoviruses

Adenoviruses (AdV) are double-stranded DNA viruses belonging to a group of non-enveloped viruses. They have a linear genome and a capsid with a classic icosahedral symmetry where hexons, pentons, and fibers that vary in length, are arranged [50]. Different serotypes are known and correlate with different tissue tropism responsible for a wide variety of clinical manifestations and syndromes such as conjunctivitis, otitis media, rhinitis, keratitis pharyngitis, gastroenteritis, and gastritis. Some adenovirus infections are asymptomatic and are responsible for the increased viral dissemination amongst the human population. Serotypes such as Ad1, Ad2, Ad3, Ad5, and Ad6 are endemics and infect mainly children; otherwise, the remaining adenovirus serotypes have an epidemic trend and can infect a large number of people [51]. The viruses are transmitted to humans via droplets generated by a cough or via the orofecal route due to the lack of adequate sanitation. The interaction between the protruding fibers and cell surface receptor, known as “CAR,” promotes the viral internalization by endocytosis mechanism [52]. The viral internalization triggers the replication of the virus, the production of viral proteins, and the modulation of cellular gene expression. In particular, adenovirus E1A and E1B proteins respectively target the tumor suppressor proteins, called Rb and p53. The E1A binds to Rb, inactivate it and enable S-phase progression, whereas E1B interacts with p53 blocking the apoptotic process which results from p53 response. Following these interactions, the infected cells are transformed to cancer cells [53]. Although adenoviruses have been identified for years, currently only two antiviral drugs, ribavirin and cidofovir, are used in anti-adenoviral therapy, but the development of drug resistance pushes the research towards new and more effective anti-adenovirus agents. The FDA has approved a vaccine preventing febrile acute respiratory disease caused by adenovirus type 4 and type 7. Although its administration is restricted to the military population. It is widely known that many natural products have biologically active compounds against viral infection. In Asian countries such as China, India, and Taiwan, medicinal plants represent the main source of antiviral drugs (Table 4).

#### 2.4.1. Natural Products Inhibiting the Capsid Protein Hexon Expression

The *Radix lithospermi* is an important Chinese medicinal plant commonly used in traditional Chinese medicine because of its anti-inflammatory, anti-tumorogenic, antibacterial property. The anti-AdV3 activity is due to a pigment, known as shikonin, extracted from the *Radix lithospermi*. The in vitro studies, performed on the HeLa cell line, discovered that shikonin inhibits the expression of hexon proteins required for the capsid formation and viral DNA packing [54].

#### 2.4.2. Natural Products Inhibiting Post-Adsorbiment Processes

*Plantago major* L. is Taiwan’s traditional medicine used in the treatment of several diseases including those caused by viral infection. Its protective role is due to five classes of biologically active compounds. Amongst phenolic compounds, the caffeic acid is active against AdV-3 (EC50 = 14.2) and *p*-coumaric acid against ADV-11 (EC50 = 43.8). Otherwise, the chlorogenic acid showed antiviral activity against AdV-3 (EC50 = 76), AdV-8 (EC50 = 108), and AdV-11 (EC50 = 13.3) and ferulic acid against AdV-8 (EC50 = 52.5) and AdV-11 (EC50 = 23.3). The real mechanism employed to interfere with viral replication is not completely understood and needs further elucidations. Preliminary data showed that caffeic acid does not inhibit the absorption of virus, rather, the viral replication of AdV3 is blocked at early stage [55]. A similar mode of action was detected by using phenolic compounds extracted by black tea (*Camellia sinensis Kuntze*) on AdV5. The treatment with *Camellia sinensis* extract led to a reduction of viral replication without interfering with the virus attachment [56]. Similarly, n-butanol fraction (EC50 = 122.5) and gallic acid (EC50 = 10.5) extracted from pomegrate peel (*Punica granatum*) as well as treatment with *Peucedanum salinum* extracts inhibited AdV5 replication in the post-adsorption phase [57] [58]. Likewise, quercetin contained in the flower of *Caesalpinia pulcherrima* Swartz and extracts from *Allium* plants prevented the early stage of viral replication and were active against serotypes 3, 8 and 41 [59,60]. Apigenin and linalool isolated from sweet basil (*Ocimum basilicum*) showed antiviral activity against AdV-3, AdV-8, and AdV-11 [61]. The real mechanism employed is not yet understood, but since apigenin is a cell cycle regulator, it is assumed that the treatment could interfere with viral replication. The characterization of natural extracts could lead to the development of potential anti-AdV agents useful as adjuvant therapy or prophylactic treatment against adenoviral infection.

## 3. Natural Compounds against RNA Viruses

### 3.1. Human Immunodeficiency Virus (HIV)

Human immunodeficiency viruses 1 and 2 (HIV-1 and HIV-2) infection leads to immunological failure and Acquired Immunodeficiency Syndrome (AIDS). HIV is a member of the lentivirus genus, which includes retroviruses that possess complex genomes. All lentiviruses are enveloped by a lipid bilayer that is derived from the membrane of the host cell. HIV-1 particles bind specifically to cells bearing the CD4 receptor, lymphocytes, and cause their destruction with a half-life of fewer than two days [62]. This leads to the fusion of HIV-1 to the host cell, thereby leading to the release of viral RNA into the cell. The reverse transcriptase enzyme presents in the virus (HIV-1) converts single-stranded viral RNA to double-stranded viral DNA. The formed viral DNA enters the host cell nucleus and incorporates the viral DNA within the host cell’s DNA by the viral enzyme integrase. This integrated viral DNA as provirus could reproduce few or no copies or remain inactive for many years. Since the introduction of highly active antiretroviral therapy (HAART), as well as the impact of preventive measures, the prevalence and incidence of HIV, have declined globally over the last decade except for parts of Eastern Europe and Central Asia [63]. However, the present therapy finds its limitations in the emergence of multidrug resistance as well as the HIV persistence within latent cellular reservoirs. Compounds derived from plant, marine, and other natural products have been found to combat HIV infection and/or target HIV reservoirs, and these discoveries have substantially guided current HIV therapy-based studies. Accordingly, finding new drugs and novel targets is needed to treat infected people and to eliminate HIV reservoirs in order to ultimately block HIV infection. Recently, several anti-HIV compounds obtained from natural products have been extensively reported [64]. However, a limited number of those are in the advanced development stage associated with well-known mechanisms of action [65] (Table 5).

#### 3.1.1. Natural Anti-HIV Compounds Directed against the Virus Entry

Griffithsin isolated from the red alga *Griffithsia sp*. is a 121 amino acid lectin that inhibits cellular entry of HIV by binding to high-mannose glycans present on the surface of the HIV envelope protein gp120 [66]. Additional lectins, such ascyanovirin-N isolated from the cyanobacterium *Nostoc ellipsosporum*, exhibit similar properties against HIV [67]. The involvement of cyclic peptides has also been identified with potent anti-HIV activity. Of these, mirabamide-A, a cyclic depsipeptide, isolated from the sponges *Siliquariaspongia mirabilis* and *Stelletta clavosa*, inhibited HIV with 50% effective concentrations (EC50s) as low as 40 nM using an in vitro model. Additional mechanistic studies suggest that the cyclic depsipeptides inhibit HIV fusion with host cell membranes during entry [68].

#### 3.1.2. Natural Inhibitors of HIV Virion Maturation

The triterpene betulinic acid, identified as an HIV inhibitor derived from the traditional Chinese medicinal herb *Syzigium claviflorum*, represent a new class of HIV therapies. Betulinic acid and dihydro betulinic acid isolated from the Chinese herb *Syzygium claviflorum* were effective at inhibiting late-stage processing the gag protein and resulted in the release of non-infectious viral particles [69]. Bevirimat, a synthetic derivative of the triterpene betulinic acid, specifically prevented cleavage of the viral capsid-spacer protein 1 intermediate, thus inhibiting HIV maturation, budding and release of viral particles and subsequent rounds of infection [70].

#### 3.1.3. Natural Reverse Trascriptase and Integrase Inhibitors

Several natural products have been identified as potent inhibitors against the enzymatic activity of HIV enzymes such as reverse transcriptase (RT) and integrase (IN). *Rheum palmatum L*, a traditional Chinese medicinal plant, also showed potent anti-RT and IN activity comparable to HAART [71]. Kuwanon-L, isolated from the black mulberry tree *Morus nigra*, has anti-RT and IN activity comparable to HAART [72]. Patentiflorin A, used in Vietnamese medicine, isolated from the plant *Justica gendarussa* had RT-inhibitor activity. The compound displayed potent inhibitory activity against drug-resistant HIV-1 isolates of both the nucleotide analogue (AZT) and nevaripine [73]. Purified calanolides, amongst the first plant-based compounds derived from *Calophyllum lanigerum* in tropical rainforests of Malaysia, demonstrated to have non-nucleoside reverse transcriptase (RT) inhibitor activity [74].

#### 3.1.4. Natural Products as Source of Latency-Reversing Agents (LRAs)

Currently licensed HIV-1 therapies highly inhibit virus replication. However, they do not act on latently infected CD4+ T cells and as a consequence they do not enable the killing and clearance of latently infected cells through immune clearance and targeting by HAART. Indeed, viral activation is required to target reservoirs of latently infected cells. One strategy to identify and eliminate the reservoirs involves a two-step process frequently termed “shock-and-kill”. Deeks and colleagues proposed the treatment of latent HIV-infected cells with latency-reversing agents (LRAs) in order to induce proviral expression (“shock”), after which these cells may be eliminated by viral cytopathic effects or host immune responses (“kill”) [75]. Many LRAs of current interest have been obtained directly from natural products. Most LRAs that have been extensively characterized to date function as either protein kinase C (PKC) activators or histone deacetylase inhibitors (HDACis) [64]. Plants of the *Euphorbia* species are the natural plant source of a third PKC agonist, ingenol [76]. Indeed, several groups have shown the efficacy of purified bryostatin, prostratin, and ingenol as LRAs in cell lines, primary CD4+ T cells, and in HIV+ HAART -suppressed patient cells in vitro. However, the toxicity of these highly effective PKC agonists limits their use. Extracts derived from *Euphorbia kansui* can reactivate latent HIV mediated by the increase of cellular CDK11 and P-TEFb with similar efficacy to purified ingenol [77]. *Theobroma cacao*, the plant source of procyanidin C1-flavonoids reactivates latent HIV through the MAPK pathway. Besides, they show synergistic activation when are present in combination with the Phorbol 12-myristate 13-acetate (PMA) a PKC agonist [78].

### 3.2. Influenza Viruses

Influenza viruses are negative-stranded, segmented RNA viruses, and are members of the *Orthomyxoviridae* family. Influenza viruses comprise three types (A, B, and C), while type A is divided into different subtypes which are distinguishable by antigenicity of their surface glycoproteins, haemagglutinin (HA), and neuraminidase (NA) [79]. Influenza viruses, based on their genetic mutations can be categorized into two different entities: seasonal or pandemic representing a major public health problem, with high rates of morbidity and mortality [80]. Airway epithelial cells lining the respiratory mucosa are the primary target of influenza infection. The recognition of influenza virus antigens through antigen-presenting cells and pattern recognition receptors (PRRs) can consequently upregulate several correspondent down-stream molecules including interleukin-6 (IL-6), IL-1β, and tumor necrosis factor α (TNF-α) which causes influenza mediated signs and symptoms [81]. To date, anti-influenza drugs include M2 ion channel inhibitors and neuraminidase inhibitors [82]. M2 ion channel inhibitors, such as amantadine and rimantadine, act as inhibitors of the uncoating process, which is essential for the release of the virus into the cytoplasm. Neuraminidase inhibitors, including oseltamivir and zanamivir, are directed against the enzymatic activity of neuraminidase, which assures the release of progeny viruses from infected cells. It has been suggested that herbal medicines might be beneficial in the prevention or management of seasonal or pandemic influenza (Table 6). An in vitro study showed that oligonol extracted from lychee fruit (*Litchi chinensis*) inhibits proliferation of influenza virus H3N2 by blocking reactive oxygen species (ROS)-dependent ERK (extracellular-signal-regulated kinases) phosphorylation [83]. Another in vitro investigation showed that green tea catechins possess higher inhibitory effects on the endonuclease activity of influenza A virus RNA polymerase [84]. Slaine and collaborators showed that the macrolide pateamine A (from *Mycale hentscheli*) and the rocagalte silvestrol (from *Aglaia*), two inhibitors of the eukaryotic initiation factor-4A (eIF4A), caused the block of influenza A viral protein synthesis as well as the failure of the viral genome replication [85]. The inhibitory effect of silvestrol was fully reversible while the pateamine A irreversibly binds to eIF4A and caused the inhibition of genetically divergent influenza A strains replication [85].

#### Natural Products as Hemagglutinin Inhibitors

Given the rapid emergence of drug resistant influenza virus strains, hemagglutinin (HA) is a promising target for developing anti-influenza drugs. HA is an envelope protein that plays a critical role in viral binding, fusion and entry processes. Curcumin showed anti-influenza activity against influenza viruses PR8, H1N1, and H6N1. The results showed more than 90% reduction in virus yield in cell culture using 30 μM of curcumin. The plaque reduction test elicited the approximate EC50 of 0.47 μM for curcumin against influenza viruses [86]. In H1N1 and also H6N1 subtypes, the inhibition of HA interaction reflected the direct effect of curcumin on infectivity of viral particles and this has proved by the time of drug addiction experiment [86]. An in vitro investigation indicated that *Cistus incanus*, a member of *Cistaceae* family, has anti-influenza virus activity in A549 (human lung epithelial cell) or MadinDarby canine kidney (MDCK) cell cultures infected with prototype avian and human influenza strains of different subtypes by the reduction of progeny virus titers of up to two logs without any toxicity [87]. Furthermore, binding of the polyphenol components of the extract to the virus surface showed protective effects through inhibition of HA binding to cellular receptors [87]. In another study, *Cistus incanus* exhibited antiviral activity against a highly pathogenic avian influenza A virus (H7N7) in both cell cultures and a mouse infection model [88]. Haidari and colleagues indicated that punicalagin from *Punica granatum* polyphenol-rich extract had anti-influenza properties in MDCK and chicken red blood cells (cRBC) infected by human influenza A (H3N2) through inhibiting the virus replication as well as inhibiting virus-induced agglutination of cRBCs [89]. Furthermore, an investigation showed that epigallocatechin gallate (EGCG) and theaflavin digallate (TF3) from green tea and black tea respectively, inhibit the infectivity of both influenza A and B viruses in MDCK cells through binding to virus HA and prevention of virus adsorption to MDCK cells [90].

### 3.3. Hepatitis C Virus

Hepatitis C virus (HCV) is an enveloped, positive-sense single-stranded RNA virus belonging to the *Flaviviridae* family. The HCV genomic RNA encodes a polyprotein that is then cleaved by both the host and virus proteases into mature proteins. The nonstructural proteins; NS2, NS3, NS4A, NS4B, NS5A, and NS5B, core proteins, glycoproteins E1, and E2; the ion channel p7 (Banerjee et al. 2010). The (NS5B), an RNA-dependent RNA polymerase (RdRp) is responsible for replicating the viral RNA genome [91]. Infected individuals have treated with standard treatment, consisting of PEGylated (PEG)-interferon (IFN)-α in combination with ribavirin (RBV) for over a decade. Recently, several protease inhibitors such as boceprevir and telaprevir have been approved as treatments for hepatitis C. However, these new inhibitors are associated with drug toxicity and the development of resistant mutants [92]. Based on this, many phytochemical constituents have been identified that display considerable inhibition of HCV life cycle at different step (Table 7).

#### 3.3.1. Natural Products Targeting Viral Entry

The absorption represents the first event in the replication cycle of HCV. This event is mediated by the interaction between E1 and E2 envelope proteins and the cellular receptor known as CD81, predominantly present on the hepatocytes surface [93]. Being the first stage in infection, viral entry is an attractive therapeutic target. To date, the standard treatment for HCV infected patients does not involve the administration of drugs targeting the viral entry mechanism. However, several natural compounds block HCV infection by interfering with the viral entry step. Three different extracts from Cameroonian medicinal plants, the roots of *Trichilia dracaena*, the stems of *Detarium microcarpum*, and the leaves of *Phragmanthera capitata* were tested on HCV infection. These extracts have the capacity to greatly interfere with the HCV entry step and are not toxic at the active concentration [94]. Likewise, *Bupleurum kaoi* extract and saikosaponins strongly inhibited HCV replication at non-cytotoxic concentrations. These natural agents targeted early steps of the viral life cycle, but had limited effect on replication, egress, and spread [95]. Data obtained by binding assays have demonstrated that delphinidin, a polyphenol plant pigment, inhibits virus entry of HCV by decreasing the amount of HCV RNA bound to the cell surface. Delphinidin affected the morphology of virus particle by acting on E1 and E2 glycoproteins inducing conformational changes on the viral particles, modifying the interaction between the viral particles and cell surface [96].

#### 3.3.2. Natural Products Targeting Viral Replication, Assembly and Release of the Virions

Several different extracts have been demonstrated to interfere with viral replication. The ethyl acetate-soluble fraction of the feather star *Alloeocomatella polycladia* was tested in cell culture on HCV replication, and this fraction exerted the strongest inhibitory effect of NS3 helicase activity [97]. The organic extract compounds derived from *Fusarium equiseti* isolated from the brown alga *Padina pavonica* were screened for their abilities to inhibit HCV NS3/4A protease (HCV PR). The derived metabolites inhibited the HCV protease (IC50 values ranging from 19 to 77 µM) [98]. *Eclipta alba* belongs to the *Asteraceae* family is used to treat infectious hepatitis in India [99]. The aqueous extract of *E. alba* and its active isolates were examined for their ability to inhibit HCV replicase (HCV NS5B) activity in vitro. The *E. alba* extract strongly inhibited the (RdRp) activity of HCV replicase in a cell culture system carrying a replicating HCV subgenomic RNA replicon. *Taraxacum officinale*, commonly known as dandelion, is frequently used in Traditional Chinese Medicine. 12 phytochemicals of *T. officinale* were selected as ligands for molecular interaction with the NS5B protein using in silico Molecular Operating Environment (MOE) software. Sofosbuvir is currently approved as a new anti HCV drug and was used as the standard in this study for comparative analysis during screening in computational docking [100]. Treatment of subgenomic replicon harboring cells with 3-hydroxy caruilignan C (3-HCL-C) isolated from *Swietenia macrophylla* stems caused a reduction of protein and RNA levels. The 3-HCL-C compound interfered with HCV replication by the induction of IFN stimulated response element transcription and IFN-dependent antiviral gene expression [101]. *Entada africana* a medicinal plant from the family *Fabaceae* is used in Africa to treat liver diseases. Galani and colleagues have demonstrated the activities of a crude *E. africana* extract and its fractions against HCV replication [102]. Grape seed extract (GSE) has been widely used as a dietary supplement because of its bioactivity properties, including antioxidant, anti-inflammatory, anti-ageing, anticancer, and antimicrobial effects [103]. The GSE treatment shows both anti-HCV activities and suppresses HCV-elevated cyclooxygenase-2 (COX-2) [104]. GSE contains large amounts of phenolic compounds, including epicatechin, quercetin, gallic acid, (þ)-catechin, dimeric procyanidin, and proanthocyanidins which display anti-HCV activity [105,106]. Nahmias and colleagues have demonstrated the dose-dependent inhibitory effect of naringenin on HCV release. The study revealed a decrease in HCV core secretion and extracellular HCV RNA-genome, demonstrating that naringenin could inhibit up to 80% HCV secretion at the concentration of 200 µM [107].

### 3.4. Picornaviruses

The family *Picornaviridae* currently contains 147 species grouped into 63 genera. Viruses in the family *Picornaviridae* have non-enveloped particles with a single-stranded RNA (ssRNA) genome and include numerous human pathogens such as poliovirus, enterovirus 71, foot and mouth disease virus (FMDV), hepatitis A virus and rhinovirus. The broadly studied and most well-characterized group is represented by enterovirus, including enterovirus A71 (EV71), coxsackievirus, poliovirus, and rhinovirus. The viral infection initiates by attaching to a receptor on the host cell plasma membrane and viruses belonging to different genera use different receptors to bind to and infect cells, thus exhibiting a different tissue tropism [108]. The antiviral drugs used in the picornaviruses treatment target virus entry (pleconaril, WIN54954 and CAR-Fc), the viral translation and/or transcription (antisense oligodeoxynucleotide and short interfering RNA) or intracellular signaling pathway (immune response activators) but do not completely eradicate the infection. To date, the antiviral activity of several natural products and herbal medicines have been tested against various *picornavirus* (Table 8).

#### 3.4.1. Broad-Spectrum Natural Antiviral Products

The orobol 7-O-d-glucoside (O7G) isolated from banaba *Lagerstroemia speciosa* L. (*Lythraceae*) was investigated for its antiviral activity against eight different strains of human rhinoviruses, cause of common viral respiratory tract infections [109]. The results showed that O7G exerts its antiviral activity on human rhinoviruses species A and species B as well as on species resistant to pleconaril. Crude aqueous and ethanolic extracts of *Ocimum basilicum* and bioactive compounds including apigenin, linalool and ursolic acid showed a strong antiviral activity towards EV71 and coxsackievirus B1 [61]. Flowers extracts from *Woodfordia fruticosa* exhibited strong anti-EV71 activity. Additionally, gallic acid isolated from *Woodfordia fruticosa* flowers possessed a strong anti-enterovirus 71 activity more than the total extracts [110]. Further in-depth studies have suggested that the content in specific secondary metabolites is responsible for the bioactive properties and therefore the antioxidant activity of gallic acid mediates its antiviral action. Raoulic acid purified from *Raoulia australis* showed a potential antiviral effect against coxsackievirus B3 and coxsackievirus B4 as well as against human rhinoviruses species A and species B [111,112].

#### 3.4.2. Natural Products Affecting Viral Components and Replication

Ursolic acid from *Ocimum basilicum* showed a strong activity against enterovirus 71 and coxsackievirus B1 in a dose- and time-dependent manner, interfering with the infection and replication phases after infection [61]. Likewise, flavonoids such as silymarin, extracted from milk thistle (*Silybum marianum*), have strong inhibition activity against enterovirus 71, showing a virucidal activity and blocking both viral attachment and entry of EV-A71 in Vero cells [113]. Besides, the extraction, fractionation and analysis of crude plant extracts of *Macaranga barteri*, normally used in Nigeria as a vermifuge, showed a high content of flavonoids in the dichloromethane (DCM) fraction, including the 3,5-dicaffeoylquinic phenolic acid (DCQA), responsible for the anti-infective activity against echoviruses E7 and E19 [114]. The molecular mechanisms employed to counteract the viral infection changes within the *Picornaviridae* family as a result of the different nature of the receptors and co-receptors used by the diverse species of picornaviruses, responsible for host interaction and entry [115]. The aqueous extract of *Syzygium brazzavillense*, a plant from the Republic of Congo, decreased the level of viral particles and viral RNA within cells infected with coxsackievirus 4 (CVB4), otherwise did not inhibit coxsackievirus A6 and enterovirus A71, coxsackievirus B1, coxsackievirus B5, belonging to the enterovirus A species, and enterovirus B [116]. The inhibition of the CVB4 replication was only obtained when the aqueous extract was incubated with the virus before adding the mixture to cell cultures, indicating that the aqueous extract can affect the stability of the capsid and interfere in the interaction between the capsid and the cellular receptor. The Chinese rhubarb including *Rheum palmatum* inhibited the coxsackievirus B3 biosynthesis without blocking viral absorption. In vitro experiments showed that the ethanol extract of rhizome and roots from *R. palmatum* decreased the viral titer and the cytopathic effect without provoking other changes in cell morphology. Likewise, in vivo studies have proven the protective efficacy of ethanol extract from *R. palmatum* on mice infected with coxsackievirus B3 and coxsackievirus B4 [117]. The CVB3-infected mice treated with the ethanol extract of R. *palmatum* showed an increased survival rate as well as alleviated clinical signs and decreased viral titers compared to the control group [117]. Quercetin-7-glucoside (Q7G) extracted from banaba leaves (*Lagerstroemia speciosa*) inhibited the human rhinovirus 2 at the early stage of viral infection with a mechanism that probably involves the indirect interaction with viral particles [118]. Likewise, it was demonstrated that the *Salvia miltiorrhiza*, known as Danshen, has a potential active ingredient, rosmarinic acid (RA) that exhibits a high inhibitory effect exclusively towards Enterovirus A71. In vitro and in vivo antiviral assays showed that rosmarinic acid did not inhibit the viral replication when pre-incubated before virus adsorption and the virus titer was not reduced when the rosmarinic acid was added at the time of virus infection. Further investigations by an attachment assay showed that the amount of bound virus, previously preincubated with rosmarinic acid, was significantly suppressed, suggesting that the rosmarinic acid affects the early stages of viral infection. The enterovirus A71-receptor binding inhibition assay revealed that rosmarinic acid exerts its antiviral activity by directly targeting virus-host receptor interaction and in particular blocking the attachment of enterovirus A71 to cellular receptors p-selectin glycoprotein ligand-1 (PSGL1) [119].

#### 3.4.3. Natural Products Targeting Host Cellular Factor

The phytochemical screening of methanol crude extract of roots from banaba *Lagerstroemia speciosa L.* (*Lythraceae*) showed the high content of alkaloids, flavonoids, saponins, tannins, and reducing sugar responsible for its antioxidant, antihypertensive, antidiabetic, antimicrobial proprieties. In particular, tannin ellagic acid from leaves of *L. speciosa* strongly inhibited RNA replication of rhinoviruses 4 in vitro only when added just after the virus inoculation but not before. This finding suggests that ellagic acid may directly interact with cellular molecules, rather than viral molecules [120]. Alternatively, other natural compounds exhibit their antiviral activity by stimulating the immune response and developing a protective state on uninfected cells. *Bupleurum kaoi* is one of the species in the *Bupleurum genus* widely known in Chinese medicine deeply studied because it exhibits its antiviral activity against coxsackievirus B1 through the induction of type I interferon in infected cells [121]. The stimulation of IFN-α and IFN-β protein expression occurs in a dose-dependent manner and reduces viral infectivity. Besides, the performed treatments, pre- and post-infection, showed that *Bupleurum kaoi* inhibited viral infection before and after viral adsorption suggesting that it may interfere with the early stage of viral infection [121]. Xiao Chai Hu Tang (XCHT) is a popular Japanese herbal drug with antiviral activity against coxsackievirus B1 at all stages of the viral life cycle. XCHT interfered with the viral adsorption at the early stage of infection exhibiting a potential prophylactic effect and likewise, induces the type I interferon expression affecting the viral replication after infection. This mechanism could arm infected and uninfected cells to counter viral replication or infection and represent an efficient therapeutic strategy [122]. An alternative strategy to counteract viral infection is to inhibit the viral genome replication by using specific competitive inhibitors which alter the formation of replication organelles, membranous structures which support the replication of the viral genome. The enteroviruses exploit the Oxysterol-Binding Protein (OSBP), as a host lipid-transport protein involved in remodelling of intracellular replication organelles [123]. Orsaponin (OSW-1) is a natural product extracted from the bulbs of the plant *Ornithogalum saundersiae* mainly employed in the anti-proliferative and anti-cancer activity [124]. Several studies demonstrated that OSW-1 binds to one of the two established OSBP ligand binding sites and induces prophylactic antiviral activity against all enteroviruses tested, enterovirus 71, coxsackievirus A21 and human rhinovirus 2 [125,126]. Antiviral protection is expressed through the reduction of intracellular levels of OSBP.

#### 3.4.4. Anti-HAV Natural Products

Genera belonging to the viral family *Picornaviridae* include hepatoviruses, such as Hepatitis A Virus (HAV). HAV seems to exclusively infect hepatocytes by interacting with the attachment host-receptor, HAVCR1, even if some studies do not identify it as an essential entry factor for HAV [127]. Unlike other *Picornaviridae*, the infection allows for the release of quasi-enveloped virions, known as eHAV, which have the same infectivity as naked virions. However, they do not have viral glycoproteins on the membrane surface, the latter being the main feature of conventional enveloped viruses. They are non-lytically secreted from infected cells as small extracellular vesicles and are found in the blood of infected patients, or the supernatant of infected cell cultures [128]. It is not clear which cell types begin the infection, which is preferentially transmitted by the fecal-oral route and rarely via blood transfusion [129]. A single serotype of human HAV is known but genetic recombination phenomena have allowed the development and the circulation of HAV genetic variants classified into six genotypes I-VI. Hepatitis A is a vaccine-preventable disease, especially where there is an endemic high prevalence of infection. Nevertheless, since preventive antiviral therapies are limited, a great effort is made to increase the safety of food products, the main reservoir of the infectious agent. To date, the employment of bioactive compounds isolated from plants could be useful to control time-temperature inactivation parameters of food-borne viruses, including hepatitis A, and ensure food safety. The epigallocatechin-3-gallate (EGCG) is catechin contained in green tea extract which has a high affinity for viral surface proteins of HAV and therefore exhibits a strong and excellent antiviral activity in pharmaceutical micro-encapsulation with chitosan and also as a natural sanitiser for foods [130]. Similarly, the anti-HAV activity of Grape Seed Extract (GSE) from *Vitis vinifera* can be used to measure the HAV levels contaminations on foods. It seems that the bioactive and antiviral properties depend on the resveratrol content. It was revealed that in vitro treatment reduces the adsorption of HAV in a dose-dependent manner, potentially blocking the cellular receptors and preventing the viral entry [131,132]. A similar mechanism is employed by blueberry proanthocyanidins which interfere with the virus-cells binding, indirectly limiting the HAV replication on cells. On the contrary, the ginsenosides extracted from *Panax ginseng* potentiate the innate immune response to HAV by activating the 2′-5′ oligoadenylate synthetase/RNase L pathway [133]. Recent studies have evaluated the anti-HAV activity of garlic (*Allium japonica*) as the most promising novel antiviral agent against HAV but further studies are necessary to clearly understand the antiviral mechanism employed to control viral infection [134].

### 3.5. Norovirus

Human noroviruses (HuNoVs) are the leading cause of gastroenteritis and severe childhood diarrhea worldwide [135]. Noroviruses belong to the family *Caliciviridae*, members of which have a small, single-stranded positive-sense RNA genome. Norovirus utilizes cell surface molecules as mediators for binding and cellular entry. Murine norovirus (MNV-1) and feline calicivirus (FCV) were successfully grown and served as a surrogate model system for HuNoV [136]. Several medicinal plants and herb extracts were screened for antiviral activity against MVN-1 and FCV as a surrogate of norovirus (Table 9). The work of Lee and colleagues showed that the components of *Morus alba* L. possess antiviral effects against foodborne enteric virus surrogates. It was found that *Morus alba* juice and its fractions inhibit the internalization and replication of MNV-1 as well as the internalization of FCV-F9 virions [137]. Black raspberry juice (*Rubus coreanus*) was found to decrease MNV-1 plaque formation by blocking viral entry into the cell and inhibiting its internalization, or through direct effects on viral particles or host cell receptors [138]. Green tea polyphenolic chatechins from *Camellia sinensis* exhibited anti-FCV-F9 antiviral activity with epigallocatechin gallate showing the best combination of antiviral activity and low cytotoxicity [139]. The essential oil from oregano (*Origanum vulgare*) decreased FCV-F9 and MNV-1 replication in a dose-dependent manner. Besides, it has been shown that oregano essential oil and its primary component carvacrol caused the loss of viral capsid integrity of MNV-1 virions as determined by transmission electron microscopy experiments [140]. Persimmon (*Diospyros kaki*) extracts containing persimmon tannin was found to reduce noroviral genome replication with no cytotoxicity effect [141].

## 4. Natural Compounds against Emerging and Re-Emerging Viruses

### 4.1. Coronaviruses

The sudden emergence of the worldwide pandemic caused by the novel coronavirus known as severe acute respiratory syndrome coronavirus 2 (SARS-CoV-2) and the lack of effective therapeutics to fight the infection and Coronavirus disease (COVID-19), emphasized for the global scientific community the need to develop antivirals or immunomodulatory drugs. Coronaviruses are enveloped RNA viruses broadly distributed among humans and other animals. Based on phylogenetic clustering, they are originally sorted into four groups: alpha and beta-coronaviruses have a mammalian host while gamma- and delta-coronaviruses infect birds. To date, seven human coronaviruses (HCoVs) have been identified. These include the α-CoVs HCoV-NL63, and HCoV-229E and the β -CoVs HCoV-OC43 and HCoV-HKU1 with low pathogenicity unlike, severe acute respiratory syndrome-CoV (SARS-CoV) and Middle East respiratory syndrome CoV (MERS-CoV) lead to potentially fatal respiratory tract infections as well as the novel SARS-CoV-2 [142,143]. The genome sequence of SARS-CoV-2 shares 96.2% of identity with bat CoV RaTG13 and 79.5% identity to SARS-CoV. For this reason, bats were thought to be the intermediary host for SARS-CoV-2 before the infection to humans [143]. To date, a large number of coronaviruses, including SARS-CoV, MERS-CoV, and more recently SARS-CoV-2 have found to have their reservoir in bats. Conversely, some other coronaviruses, such as the HCo-OC43, have their primary host in rodents. In some cases, the intermediate host for HCoV has been identified. For SARS-CoV the intermediate host is masked palm civet, whereas for MERS-CoV the intermediate host is dromedary camel [144]. Human-to-human contamination prevalently occurs via respiratory droplets and aerosols. The pathogenesis of HCoVs initiates with the entry of virus particles mediated by structural protein Spike (S protein) of HCoVs. The S1 subunit of Spike protein contains the receptor-binding domain (RBD) and binds to the cellular receptor and the S2 subunit promotes the fusion and entry. This is a critical step for the infection and depends on cells susceptibility defined by cellular receptors localized on target cells. To date, except for HCoV-HKU1, cellular receptors have been identified for all HCoVs: ACE2 is the receptor for SARS-CoV, Dipeptidyl peptidase 4 (DPP4, also known as CD26) is the receptor for MERS-CoV, Human aminopeptidase N (CD13) is the receptor for HCoV-229E and the 9-O-acetylated sialic acid is the receptor for HCoV-OC43 [142,145]. The SARS-CoV-2 virus has more sophisticated cell entry mechanisms based on the binding between spike-RBD and hACE2 and on the activation of cellular proteases which induce a structural change of S2 needed for the membrane fusion [146]. Therefore, knowledge about cellular recognition receptor is important not only for the pathogenesis but also for the development of intervention strategies towards viral infection. Several antiviral drugs treatments are under investigation, including inhibitors of viral entry by targeting ACE2 receptor or the transmembrane serine protease 2 (TMPRSS2), an inhibitor of viral membrane fusion, or inhibitor of the SARS-CoV-2 RNA-dependent RNA polymerase [147]. To date, Remdesivir is the only Food and Drug Administration (FDA)-approved drug for the treatment of COVID-19 in hospitalized patients Remdesivir is a nucleotide analogue of adenosine, which act as an inhibitor of the SARS-CoV-2 RNA-dependent RNA polymerase. Once phosphorylated into the cells, Remdesivir competes with the adenosine triphosphate (ATP) for incorporation into nascent RNA chains and cause premature termination of RNA transcription [148,149]. However, although a positive effect in terms of time to recovery, clinical treatment with the remdesivir alone is not likely to be sufficient for all patients. Currently, combinatory drug approaches, as well as SARS-CoV-2 monoclonal antibodies and immune-modulatory drugs treatments, are in development as post-exposure preventive strategies. Besides, other therapeutic choices such as plant-based natural compounds are considering as a rich resource for novel antiviral drug development against coronaviruses [150,151] (Table 10).

#### 4.1.1. Natural Products Targeting Viral Proteins

The high similarity between SARS-CoV-2 and SARS-CoV allows using of existing MERS and SARS inhibitors for COVID-19 treatment. The spike glycoprotein represents the therapeutic target of natural compounds such as emodin extracted from *Rheum officinalis* [152], ginsenoside-Rb1 extracted from *Panax ginseng* [153], secomet-V extracted from *African trifolium* [154] and tetra-O-galloyl-β-D-glucose extracted from *Galla chinensis* to SARSCoV [155], saikosaponin B2 extracted from *Bupleurum chinense* [156] to HCoV-229E. The bisbenzylisoquinoline alkaloids-tetrandrin extracted from *Stephaniae tetrandrae* radix has been found to inhibit viral S and N protein expression of the HCoV-OC43 [157].

#### 4.1.2. Natural Products Targeting the 3CLpro

Promising candidates in the treatment of SARS-CoV target the coronavirus main proteinase-chymotrypsin-like protease (3CLpro), an essential player in viral replication. The proteolytic cleavage of polyprotein at specific sites by 3CL protease is essential for the replication of SARS-CoV and is used to monitor the anti-enzymatic activity of compounds. Quercetin, a flavonoid extracted from *Ginkgo biloba*, affects SARS-CoV-2 3CLpro stability, interacts with and binds to 3CLpro active site [158]. Likewise, triterpenes such as celastrol, pristimerin, tingenone, and iguesterin isolated from *Triterygium regelii* showed an inhibitory effect on SARS-CoV-2 3CLpro. The reduction of enzymatic activity of 3CLpro was attributed to the presence of a quinone-methide moiety, which seems to enhance the inhibition. Computer docking analysis has also revealed that iguesterin fits in the substrate-binding pocket of 3CLpro suggesting that it can to be a competitive inhibitor for protease active site [159]. Several known phytochemicals were tested for inhibitory effects on SARS-CoV 3CLpro though the protease inhibition assay. Terpenoids, lignoids, curcumin, niclosamide, and valinomycin were able to induce high inhibition activity with differences of intermolecular interaction due to chemical structure. In particular, the triterpenes betulinic acid and the lignoid savinin, unlike to lignoid hinokinin, form single and double hydrogen bonding with the specific amino acid residues located at the active site pocket of the enzyme. This interaction strengthens and ensures the binding with 3CLpro [160]. Likewise, the presence of a gallate group on black tea polyphenols such as the 3-isotheaflavin-3-gallate (TF2B) and theaflavin-3,3’-digallate (TF3) seems to be responsible for the inhibitory activity against 3CLpro [161]. A pool of compounds extracted from *Isatis indigota* root including phenolic compounds were tested for anti-SARS-CoV 3CLpro. Only sinigrin, indigo, aloe emodin and hesperetin inhibited the cleavage activities of the 3CLpro demonstrating the significant inhibitory effects on enzyme [162].

#### 4.1.3. Broad-Spectrum Antiviral Activity of Curcumin against SARS-CoV-2

Recently, current knowledge on the pleiotropic effects of curcumin on the capability to inhibit viral entry and the viral protease and modulate the intracellular signaling pathways in viruses belonging to different families has been comprehensively discussed [163]. The effect of curcumin on viral replication was previously tested using a cell-based assay measuring SARS-CoV-induced cytopathogenic effect on Vero E6 cells [160]. Since curcumin is known to have strong inhibitory effects on the expression of various pro-inflammatory cytokines responsible for the "cytokine storm" that occurs in serious cases of COVID-19, in silico docking studies have recently supported the potential role of curcumin as a promising antiviral agent against SARS-CoV-2 infection [163].

#### 4.1.4. Natural Products Targeting the Host Protein Synthesis Machinery

Host proteins are an important factor for the life cycle of many viral pathogens. Therefore, inhibition of viral replication by targeting the host proteins is a promising therapeutic approach in the treatment of viral infection. Silvestrol, a member of rocaglates family isolated from *Aglaia* plants, has been demonstrated to target the eukaryotic initiation factor-4A (eIF4A), an RNA helicase which activity is required to unwind RNA secondary structures in the 5’ untranslated region (5′-UTRs) and facilitate translation initiation [164,165]. Müller and collaborators demonstrated that silvestrol inhibited cap-dependent viral mRNA translation of MERS-CoV and HCoV-229E in human embryonic lung fibroblast (MRC-5) cells with EC_50_ value of 1.3 nM and 3 nM, respectively [166]. Furthermore, in peripheral blood mononuclear cells (PBMCs) silvestrol inhibits the expression of MERS-CoV structural and nonstructural proteins (N, nsp8) and the formation of viral replication/transcription complexes [166]. Additional reports showed that a synthetic rocaglate CR-31-B (-) possesses antiviral activity against HCoV-229E, similar to silvesterol, and SARS-CoV-2 replication in both in vitro and ex vivo studies [167,168]. Since, to date, chemical synthesis of silvestrol remains difficult, alternative synthetic rocaglate such as the CR-31-B (-) are desirable and promising anti-coronaviruses agents. Hippuristanol, a polyhydroxysteroid isolated from *Isis hippuris*, is another eIF4A inhibitors that prevent its binding to RNA and reduce HCoV-229E replication, similarly to silvesterol [169]. Plitidepsin is a cyclic depsipeptide isolated from *Aplidium albicans*, a Mediterranean marine tunicate. Plitidepsin is synthesize and commercialized as Alpidin, a limited licensed drug for the treatment of multiple myeloma and its therapeutic target is the eukaryotic translation elongation factor 1A (eEF1A) [170]. This cellular factor is required for the enzymatic delivery of aminoacyl tRNAs to the ribosome and it has been previously described to be an important host factor for the replication of many viral pathogens, including respiratory syncytial virus and gastroenteritis coronavirus [171,172]. An in vitro and in vivo study conducted by White and collaborators showed that plitidepsin possesses antiviral activity against SARS-CoV-2 through inhibition of eEF1A [173]. Plitidepsin was found to be more effective in vitro than remdesivir by a factor of 27.5 in Vero E6 cells (IC_50_ = 0.70 nM, CC_50_ = 1.99 nM), in human cell line hACE2-293T (IC_50_ = 0.73, CC_50_ ≥ 200 nM) as well as in pneumocyte-like cells (IC_50_ = 1.62, CC_50_ = 65.43 nM) [173]. The in vivo efficacy was demonstrated in two mouse models of SARS-CoV-2 infection, indicating that plitidepsin treatment reduces the replication of SARS-CoV-2 as well as lung inflammation [173]. Additionally, it has been reported that plitidepsin retained its efficacy against the new SARS-CoV-2 B.1.1.7 variant [174]. A phase I/II clinical study has also been completed for the use of plitidepsin in the treatment of COVID-19 [175].

### 4.2. Flaviviruses

Flaviviruses are enveloped, single-stranded RNA viruses belonging to the family *Flaviviridae*. The genus *Flavivirus* contain viruses that are transmitted by either mosquitoes or ticks (arthropod-borne) [176]. The *Flavivirus* genus contains important pathogens such as Zika virus (ZIKV), Dengue virus (DENV), yellow fever virus (YFV), Japanese encephalitis virus (JEV), and West Nile virus (WNV) among others. Flaviviruses are generally 50 nm in diameter and have icosahedral symmetry. The core of the virus is the nucleocapsid, which contains a single-stranded, non-segmented, positive polarity 11-Kb genome along with structural C proteins [176]. Host cells for flaviviruses infection include monocytes, macrophages and dendritic cells. Natural compounds and crude plant extracts possess antiviral activity against various Flaviviruses (Table 11). It is of particular interest to note that flavonoids have been shown to possess antiviral activity. Compounds like rutin, quercetin, and ellagic acid have been found to inhibit DENV-2 virus replication in C6/36 cells [177]. Other studies showed that epigallocatechin (EGC), epigallocatechin gallate (EGCG) and Delphinidin chloride (D) inhibit WNV infection while the last two also inhibit DENV and ZIKV. Thereby, quantitative RT-PCR showed that both polyphenols significantly reduced the amount of viral RNA in the supernatant of infected cultures [178]. The galactomannans from *Mimosa scabrella* (BR) and *Leucaena leucocephala* (LL) have shown good inhibitory actions on YFV (BeH111 strain) and DENV-1 (Hawaii strain) [179]. In addition, xindole alkaloids from *Uncaria tomentosa* induced apoptosis, favoring the elimination of virus-infected cells. In vitro studies on DENV-2 infected human monocytes revealed their IFN-α inhibitory effect [180]. Other polyphenols have shown similar mechanisms of action, such as baicalein that exhibited anti-adsorption and virucidal activities against DENV-2 and JEV [181], and quercetin that presented anti-adsorption and anti-attachment activity against DENV-2 [182]. Studies have revealed that the inhibitory effect of the aqueous extract of *Azadirachta indica* leaves on DENV-2 is due to suppression of viral entry and reduction in viral replication [183]. Furthermore, it has been reported that curcumin may directly neutralize ZIKV infection by inhibiting the attachment of virus infection [184]. Previously, curcumin was shown to potentially inhibit DENV-2 infection through direct effects on viral particle production or various cellular systems [185]. The effects of silvestrol (from *Aglaia* plant), a known inhibitor of the eIF4A helicase, were assessed on ZIKV replication. The results showed that silvestrol inhibits the replication of two different ZIKV strain in both A549 cells and primary human hepatocytes [186]. The potential antiviral activity of silvesterol against ZIKV suggest that the eIF4A could represent a cellular target to inhibit ZIKV spread.

### 4.3. Togaviruses

Chikungunya virus (CHIKV) is a mosquito-transmitted alphavirus belonging to the family *Togaviridae* [187]. It was isolated for the first time from a Tanzanian outbreak in 1952. It is responsible for an acute infection of abrupt onset, characterized by high fever, arthralgia, myalgia, headache, and rash. CHIKV is an enveloped, positive-strand RNA virus. To date, two CHIKV complete nucleotide sequences have been determined, for the strains Ross and S27 [188]. Four nonstructural proteins (nsP1-nsP4) are encoded by the 5’ ends of the genome. The 3’ end of the genome encodes a polyprotein precursor containing three structural proteins: PE2 (the precursor of E3 and E2), E1 and the capsid protein (C) [189]. Many natural compounds showed antiviral activity against a variety of human viruses such as CHIKV (Table 11). Epigallocatechin gallate (EGCG) has shown to possess antiviral activity against CHIKV in vitro through the inhibition of virus entry to target cells [190]. Furthermore, the flavonoids baicalein, fisetin, and quercetagetin showed anti-CHIKV activity by inactivating the virus, preventing the attachment of the virus to the host cells and blocking post-entry stages [191]. Berberine is a compound found in plants from the *Berberidaceae* family, showed decreased viral RNA and viral protein synthesis, suggesting that berberine is indirectly perturbing CHIKV replication by affecting host components [192].

### 4.4. Filoviruses

The Ebola virus (EBOV) is amongst the most pathogenic viruses known to cause viral hemorrhagic fever outbreaks in Africa. EBOV virus is enveloped, negative-sense RNA genome belonging to the *Filoviridae* family [193]. The genomic RNA of approximately 19 kb is encapsidated by the nucleoprotein and, together with polymerase L, transcription activator VP30, and polymerase cofactor virion protein (VP) 35, constitutes the nucleocapsid. It encodes seven distinct genes from which, using RNA editing, at least nine proteins are expressed: nucleoprotein (NP), transcription activator (VP30), polymerase cofactor (VP35), matrix protein (VP40), glycoprotein (GP), soluble GP (sGP), minor matrix protein (VP24), small soluble GP (ssGP), and RNA-dependent RNA polymerase (L) [194]. EBOV causes severe hemorrhagic fever in humans, with human case fatality rates of up to 90%. Since the 2014 EBOV outbreak in West Africa responsible for over 11,000 deaths in ten countries, no vaccine or drug has been approved [195]. Recent research suggests that plant-derived natural products and structurally similar small molecules are worthy of closer inspection [196]. In particular, seven small natural compounds were selected for testing against EBOV [197]. Of these, eugenol and *p*-anisaldehyde were selected based on their commercial availability and previous testing for biological activity and displayed anti-EBOV activity [197] (Table 11). The rocaglate silvestrol, isolated from *Aglaia* plant, is known to specifically inhibit the eIF4A helicase. Biedenkopf and collaborators showed that silvestrol inhibits EBOV replication in human primary macrophages at low nanomolar concentrations [198]. Furthermore, the antiviral activity of silvesterol correlates with the shutdown of EBOV proteins synthesis as well as with a reduced expression of the proto-oncoprotein PIM1, a cellular kinase that negatively affects cell proliferation and thereby also viral propagation [198,199]. Further efforts to characterize this natural product in EBOV should be done to better understand the mechanisms of action.

## 5. Discussion

Despite the increased advancement in providing effective antiviral treatments, several problems exist with the currently available antiviral drugs, including high costs, drug-resistance phenomena, safety, and efficacy limitations [200]. Indeed, the peculiarity of viral replication gives rise to the related side effect of antiviral drugs since it is difficult to attack the virus without affecting the host cells. Additionally, the high viral variability, especially RNA viruses, and accumulated mutation in the viral genome, are responsible for the emergence of drug-resistant viruses, which are challenging many researchers for the design of novel antiviral drugs.

Natural products represent a rich source for novel antiviral compounds. Indeed, over the past years considerable advancements have been made for the identification of their antiviral mechanisms. A large number of natural products, including polysaccharides, polyphenols, anthocyanins, and phenolic compounds, have been reported to have potential in vitro and in vivo antiviral activities in pre-clinical studies and some have been included in clinical trials for drug development [201,202]. Unlike combinatorial synthetic drugs, these natural active compounds have high biochemical specificity against a wide range of molecular targets while remaining capable of being absorbed and metabolized with low toxic effect. Another serious problem in viral pathogenesis is represented by emerging and re-emerging viruses, which have resulted in many outbreaks, epidemics, and pandemics, includingthe current emergence of COVID-19 disease due to the worldwide spread of SARS-CoV-2, affecting both social and economic conditions worldwide [203]. In conclusion, although large numbers of natural compounds have been isolated from medicinal plants, only few have been marketed as pharmaceutical products. Thus, further characterization of the active compounds will be useful for the treatments of viral infections and the management of pandemic outbreaks. 

## Figures and Tables

**Table 1:** Natural compounds and their antiviral targets against Hepatitis B virus.

Natural Source	Compound	Hepatitis B Virus	Target	CC_50_	IC_50_	SI	Reference
*Terminalia bellerica*	Methanolic extracts	Hepatitis B virus	Inhibits viral entry, replication and maturation of HBV particles				[15,16]
*Enicostemma axillare*	Methanolic extracts	Hepatitis B virus					
*Phyllanthus Amarus*	Crude extract	Hepatitis B virus					
*Hybanthus enneaspermus*	Methanolic extracts	Hepatitis B virus					
*Bombyx mori* L.	Iminosugars 1-deoxynojirimycin (1-DNJ)	Hepatitis B virus		>50 mM	2.96 mM	208	[17]
*Boehmeria nivea*	Chloroform fraction Ethyl acetate fraction	Hepatitis B virus			19.67–20.92 mg/L 36.45–39.90 mg/L		[18]
*Pulsatilla chinensis*	Betulinic acid	Hepatitis B virus	Targeting host cellular factor				[19]
*Curcuma longa L.*	CLL extract	Hepatitis B virus					[20]
*Liriope platyphylla*	LPRP-Et	Hepatitis B virus					[21]

CC_50_: Half maximal cytotoxic concentration; IC_50_: Half maximal inhibitory concentration; SI: Selectivity index = CC_50_/IC_50_.

**Table 2:** Natural compounds and their antiviral targets against Herpesviruses.

Natural Source	Compound	Herpesvirus	Target	CC_50_	IC_50_	SI	Reference
*Curcuma longa* L	Curcumin Gallium-curcumin Cu-curcumin	Herpes simplex virus-1	Inhibits immediate-early gene expression	484.2 μg/mL 255.8 μg/mL 326.6 μg/mL	33.0 μg/mL 13.9 μg/mL 23.1 μg/mL	14.6 18.4 14.1	[25,26]
*Houttuynia cordata*	Quercetin	Herpes simplex virus-1 Herpes simplex virus-2	Blocks viral binding and suppresses NF-kB activation				[30]
*Pistacia vera* L	Polyphenols-rich exstract	Herpes simplex virus-1	Inhibits expression of HSV-1 viral proteins and viral DNA synthesis	1.2 mg/mL	0.4 mg/mL	3	[27]
*Prunus dulcis*	Polyphenols-rich exstract	Herpes simplex virus-1	Blocks viral binding	0.6 mg/mL			[31,32]
*Aloe Vera*	Polyphenols-rich exstract	Herpes simplex virus-1	Reduction of cytopathic effect (CPE)				[28]
*Cephalotaxaceae*	Homoharringtonine	Herpes simplex virus-1	Targets the cellular factor eIF4E		139 nM		[34]

CC_50_: Half maximal cytotoxic concentration; IC_50_: Half maximal inhibitory concentration; SI: Selectivity index = CC_50_/IC_50_.

**Table 3:** Natural compounds and their antiviral targets against Papillomaviruses.

Natural Source	Compound	Papillomaviruses	Target	Reference
*Curcuma longa L.*	Curcumin	HPV-16 HPV-18	Downregulating expression of oncogenes E6 and E7	[39]
*Ficus carica*	Latex	HPV-16		[41]
*C. longa, A. indica*, *E. officinalis*, *A. vera*	Basant	HPV-16	Prevents the entry of HPV 16 in Hela cells	[40]
*Green tea*	Epigallocatechin-3-gallate (EGCG)	HPV-16	Promoting apoptosis and inhibiting cellular transcriptional factors	[43]
*Pinellia pedatisecta*	Rhizome extract	HPV-16 HPV-18		[44]
*Cudrania tricuspidata*	Stem extract	HPV-16		[45]
*Curcuma longa L.*	Curcumin	HPV-18 HPV-16		[46]
*Bryophyllum pinnata*	Leaves extract	HPV-18		[47]
*Phyllanthus emblica*	Crude extract	HPV-16 HPV-18		[48]
*Kaempferia parviflora*	Ethanolic extract	HPV-16		[49]

**Table 4:** Natural antiviral compounds against Adenoviruses.

Natural Source	Compound	Adenoviruses	Target	CC_50_ (µg/mL)	EC_50_-IC_50_ (µg/mL)	SI	Reference
*Radix Lithospermi*	Shikonin	AdV3	Downregulation of capsid protein hexon expression				[54]
*Plantago major L.*	Caffeic acid	AdV-3	Inhibition of post-adsorbiment processes		14.2	727	[55]
*Plantago major L.*	p-coumaric acid	AdV-11			43.8	11.2	
*Plantago major L.*	Chlorogenic acid	AdV-3 AdV-8 AdV-11			76 108 13.3	52.6 36.9 301	
*Plantago major L.*	Ferulic acid	AdV-8 AdV-11			52.5 23.3	1.8 4	
*Camellia sinensis Kuntze*	Phenolic compounds	AdV-5		165.95 ± 12.7	6.62 ± 1.4	25.06	[56]
*Punica granatum*	n-butanol fraction	AdV-5		264.7	2.16	122.5	[57]
*Punica granatum*	Gallic acid	AdV-5		49.34	4.67	10.5	
*Peucedanum salinum*	Methanol extract	AdV-5					[58]
*Peucedanum salinum*	Methanol/H2O extract	AdV-5					
*Caesalpinia* *pulcherrima Swartz*	Quercetin	AdV-3 AdV-8 AdV-41		496.9	24.3 ± 4.9 39.9 ± 5.4 44.8 ± 9.4	20.4 12.5 11.1	[59]
*A. ascalonicum L.*	Crude aqueous extract	AdV-41 AdV-3		2696.8 2696.8	733.9 1137.60	3.7 2.4	[60]
*A. sativum L.*	Crude aqueous extract	AdV-41 AdV-3		2649.6 2649.6	3500 3500		
*A. fistulosum L.*	Crude aqueous extract	AdV-41 AdV-3		>960 >960	>960 >960		
*Ocimum basilicum*	Apigenin	AdV-3 AdV-8 ADV-11		59.9	11.1 ± 0.9 8 ± 0.8 20.9 ± 0.1	5.4 7.5 2.9	[61]
*Ocimum basilicum*	Linalool	AdV-3 AdV-8 ADV-11		177.1	24.4 ± 0.4 26.4 ± 0.6 16.9 ± 0.3	7.3 6.7 10.5	
*Ocimum basilicum*	Ursolic acid	AdV-8 ADV-11		100.5	4.2 ± 0.3 24.5 ± 0.3	23.8 4.1	

CC_50_: Half maximal cytotoxic concentration; EC_50_-IC_50_: Half maximal inhibitory concentration; SI: Selectivity index = CC_50_/IC_50_.

**Table 5:** Natural compounds and their antiviral targets against Immunodeficiency virus.

Natural Source	Compound	Immunodeficiency Virus	Target	CC_50_	EC_50_-IC_50_	SI	Reference
*Griffithsia* sp.	Griffithsin	HIV	Entry inhibitors		0.043–0.63 nM		[66]
*Nostoc ellipsosporum*	Ascyanovirin-N	HIV					[67]
*Siliquariaspongia mirabilis* *Stelletta clavosa*	Mirabamide-A	HIV			40–140 nM		[68]
*Syzygium claviflorum*	Betulinic acid Dihydro betulinic	HIV	Maturation inhibitors		1.4 µM 0.9 µM	9.3 14	[69]
Synthetic derivative of betulinic acid	Bevirimat	HIV		25 μM	7.8 nM	>2500	[70]
*Rheum palmatum*	Sennoside A	HIV	Reverse trascriptase and Integrase inhibitors				[71]
*Morus nigra*	Kuwanon-L						[72]
*Justica gendarussa*	Patentiflorin A	HIV			24−37 nM		[73]
*Calophyllum lanigerum*	Calanolides	HIV			0.1–0.4 µM		[74]
*Euphorbia kansui*	Ingenol Bryostatin Prostratin	HIV	Latency-reversing agents (LRAs)				[77]
*Theobroma cacao*	Procyanidin C1-flavonoids	HIV					[78]

CC_50_: Half maximal cytotoxic concentration; EC_50_-IC_50_: Half maximal inhibitory concentration; SI: Selectivity index = CC_50_/IC_50_.

**Table 6:** Natural products against Influenza viruses.

Natural Source	Compound	Influenza Viruses	Target	CC_50_	EC_50_-IC_50_	SI	Reference
*Litchi chinensis*	Oligonol	H3N2	Blocking (ROS)-dependent ERK phosphorilation				[83]
Green tea	Catechins	H1N1	Inhibiting RNA polymerase				[84]
Aglaia	Silvestrol	H1N1	Inhibitors of the cellular factor eEIF4A				[85]
*Mycale hentscheli*	Pateamine A	H1N1 H3N2					
*Curcuma longa L.*	Curcumin	H1N1 H6N1	Haemagglutinin inhibitors	43 µM	0.47 µM	92.5	[86]
*Cistus incanus*	Polyphenol rich extract	A549			50 μg/mL		[87]
*Punica granatum*	Punicalagin	H3N2					[89]
Green tea	Epigallocatechin gallate	H1N1					[90]
Blak tea	Theaflavin digallate	H1N1					

CC_50_: Half maximal cytotoxic concentration; EC_50_-IC_50_: Half maximal inhibitory concentration; SI: Selectivity index = CC_50_/IC_50_.

**Table 7:** Natural products against Hepatitis C virus.

Natural Source	Compound	HCV	Target	CC_50_ (µg/mL)	EC_50_-IC_50_ (µg/mL)	SI or TI	Reference
*Trichilia dregeana*	Root extract	HCV	Inhibition of viral entry		16.6	37	[94]
*Detarium* *microcarpum*	Stem bark extract				1.42	211	
*Phragmanthera* *capitata*	Leave extract				13.17		
*Bupleurum kaoi*	Saikosaponin B2			740.4 ± 28.35 µM	16.13 ± 2.41 µM	45.9	[95]
*Bupleurum kaoi*	Methanolic extract			16.82 ± 1.89	215.4 ± 10.7	12.8	
Anthocyanidin	Delphinidin				3.7 ± 0.8 μM		[96]
*Alloeocomatella polycladia*	Ethyl acetate- soluble fraction		Suppression of the helicase activity of HCV NS3		11.7 ± 0.7		[97]
*Fusarium equiseti*	Crude extracts		Inhibition of HCV NS3/4A protease		19–77 µM		[98]
*Eclipta alba*	Aqueous extract		Inhibition of HCV NS5B replicase activity		11		[99]
*Taraxacum officinale*	Flavonoids						[100]
*Swietenia macrophylla*	3-hydroxy caruilignan C		Reduction of HCV protein and HCV- RNA levels		10.5 ± 1.2μM		[101]
*Entada africana*	Methylene chloride-methanol (MCM) stem bark crude extract		Broad antiviral activity		453 ± 0.00117		[102]
*Grape seed*	Phenolic compounds		Suppression of HCV- induced Cox-2		7.5 ± 0.3		[104]
*Flavanone*	Naringenin		Release/Assembly		109 μM		[107]

CC_50_: Half maximal cytotoxic concentration; EC_50_-IC_50_: Half maximal inhibitory concentration; SI: Selectivity index = CC_50_/IC_50_.

**Table 8:** Natural compounds and their antiviral targets against Picornaviruses.

Natural Source	Compound	Picornaviruses	Target	CC_50_ (µg/mL)	EC_50_-IC_50_ (µg/mL)	SI	Reference
*Lagerstroemia* *speciosa* L.	Orobol 7-O-d-glucoside (O7G)	Human rhinovirus A Human rhinovirus B	Broad spectrum antiviral activity	100	0.58-8.80	12	[109]
*Ocimum basilicum*	Crude aqueous extracts	Coxsackievirus B1 Enterovirus 71		1469.3	105.7 ± 2.6 200.2 ± 3.2	13.9 7.3	[61]
*Ocimum basilicum*	Ethanolic extracts	Coxsackievirus B1 Enterovirus 71		684.8	146.3 ± 2.9 198.9 ± 1.8	4.7 3.4	
*Woodfordia fruticosa*	Gallic acid	Enterovirus 71		100	0.76	132	[110]
*Raoulia australis*	Raoulic acid	Human rhinovirus 2 Human rhinovirus 3 Coxsackievirus B3 Coxsackievirus B4 Enterovirus 71		201.78 65.86	0.1 0.19 0.33 0.40 0.1		[111,112]
*Ocimum basilicum*	Ursolic acid	Coxsackievirus B1 Enterovirus 71	Targets viral structures and inhibits viral infection and replication process	100.5	0.4 ± 0.1 0.5 ± 0.2	251 201	[61]
*Sylibum marianum*	Silymarin	Enterovirus 71		160.20 ± 1.56	7.99 ± 3.0	20.05	[113]
*Macaranga barteri*	DCM fraction	Echoviruses E7 Echoviruses E19		0.18	7.54 × 10^−6^ 1.75 × 10^−6^	19.9 8581.24	[114]
*Syzygium* *brazzavillense*	Aqueous extract	Coxsackievirus B4		2800	0.8		[116]
*Rheum palmatum*	Ethanol extract	Coxsackievirus B3			4	10	[117]
*Lagerstroemia* *speciosa L.*	Quercetin-7-glucoside (Q7G)	Human rhinovirus 2		>100	4.85–0.59	>20.62	[118]
*Salvia miltiorrhiza*	Rosmarinic acid	Enterovirus A71		327.68 ± 14.43	31.57–114	2.87–10.36	[119]
*Green tea*	Epigallocatechin-3-gallate (EGCG)	Hepatitis virus A					[130]
*Vitis vinifera*	Grape seed extract (GSE)	Hepatitis virus A					[131,132]
*Lagerstroemia* *speciosa* L.	Tannin ellagic acid	Human rhinovirus 2 Human rhinovirus 3 Human rhinovirus 4	Targets host cellular factors	>100	38 ± 3.2 31 ± 5.2 29 ± 2.5	>2.6 >3.2 >3.4	[120]
*Bupleurum kaoi*	Roots extract	Coxsackievirus B1		883.56	50.93		[121]
Mix of seven medicinal herbs	Xiao chai hu tang	Coxsackievirus B1		945.75	50.93	18.92	[122]
*Ornithogalum* *saundersiae*	Orsaponin (OSW-1)	Enterovirus 71, Coxsackievirus A21 Human rhinovirus 2		>100 nM	2.4–9.4 nM		[123,124]
*Panax ginseng*	Ginsenosides	Hepatitis virus A					[133]

CC_50_: Half maximal cytotoxic concentration; EC_50_-IC_50_: Half maximal inhibitory concentration; SI: Selectivity index = CC_50_/IC_50_.

**Table 9:** Natural products against Noraviruses.

Natural Source	Compound	Noroviruses	Target	CC_50_	EC_50_-IC_50_	SI	Reference
*Morus alba L*	Juice	MNV-1 FCV-F9	Inhibiting internalization and replication	>0.1% >2.5%	0.005% 0.25%	20 10	[137]
*Camellia sinensis*	Epigallocatechin gallate	FCV-F9			12 mg/mL		[139]
*Rubus coreanus*	Juice	MNV-1					[138]
*Origanum vulgare*	Carvacrol	MNV-1					[140]
*Diospyros kaki*	Persimmon tannin	HuNoV	Reduce genome replication				[141]

CC_50_: Half maximal cytotoxic concentration; EC_50_-IC_50_: Half maximal inhibitory concentration; SI: Selectivity index = CC_50_/IC_50_.

**Table 10:** Natural compounds and their antiviral targets against Coronaviruses.

Natural Source	Compound	Coronaviruses	Target	CC_50_	EC_50_-IC_50_	SI	Reference
*Rheum officinalis*	Emodin	SARSCoV	Targeting viral proteins		200 μM		[152]
*Panax ginseng*	Ginsenoside-Rb1	SARSCoV					[153]
*African trifolium*	Secomet-V	SARSCoV					[154]
*Galla chinensis*	Tetra-O-galloyl-β-D-glucose	SARSCoV		1.08 mM	4.5 μM	240	[155]
*Bupleurum chinense*	Saikosaponin B2	HCoV-229E		383.3 ± 0.2 µmol/L	1.7 ± 0.1 µmol/L	221.9	[156]
*Stephaniae tetrandrae*	Bisbenzylisoquinoline alkaloids-tetrandrin	HCoVOC43		14.51 μM	295.6 nM	>40	[157]
*Ginkgo biloba*	Quercetin	SARS-CoV-2	3CLpro inhibitory activity				[158]
*Triterygium regelii*	Triterpenes	SARS-CoV-2			2.6–10.3 μM		[159]
Black tea	3-isotheaflavin-3-gallate (TF2B) and theaflavin-3,3’-digallate (TF3)	SARS-CoV-2			≤10 μM		[161]
*Isatis indigota*	Sinigrin Hesperetin	SARS-CoV		>10,000 μM 2718 μM	217 μM 8.3 μM		[162]
*Curcuma longa L.*	Curcumin	SARS-CoV-2	Broad-spectrum activity				[160,163]
*Aglaia* genus	Silvestrol	MERS-CoV HCoV-229E	Inhibitors of viral mRNA translation		1.3 nM 3 nM		[166]
	CR-31-B (-)	HCoV-229E SARS-CoV-2					[167,168]
*Isis hippuris*	Hippuristanol	HCoV-229E					[169]
*Aplidium albicans*	Plitidepsin	SARS-CoV-2		1.99 −> 200 nM	0.70–1.62 nM		[173]

CC_50_: Half maximal cytotoxic concentration; EC_50_-IC_50_: Half maximal inhibitory concentration; SI: Selectivity index = CC_50_/IC_50_.

**Table 11:** Natural products against Flaviviruses, Togaviruses, and Filoviruses.

Natural Source	Compound	Flavivirus	Target	CC_50_ (µg/mL)	EC_50_-IC_50_ (µg/mL)	SI	Reference
*Spondia mombin* *Spondia tuberosa*	Rutin Quercetin	DENV-2	Broad spectrum antiviral activity	<1000	362.68 500	2.75 2	[177]
	Epigallocatechin Epigallocatechin gallate Delphinidin chloride	WNV DENV ZIKV					[178]
*Mimosa scabrella* *Leucaena leucocephala*	Galactomannans	YFV DENV-1					[179]
*Uncaria tomentosa*	Xindole alkaloids	DENV-2	Immunomodulatory effects				[180]
	Baicalein	DENV and JEV	Inhibits the virus attachment	115.2 ± 0.2	3.4–5.8	1.3–33.4	[181]
	Quercetin	DENV-2			28.9–35.7	7.07–8.74	[182]
*Azadirachta indica*	Aqueous extract	DENV-2					[183]
*Curcuma longa*	Curcumin	ZIKV DENV-2					[184,185]
*Aglaia*	Silvestrol	ZIKV	Target the cellular factor eIF4A				[186]
**Natural source**	**Compound**	**Togaviruses**	**Target**	**CC_50_** **(µg/mL)**	**IC_50_ (µg/mL)**	**SI**	**Reference**
Green tea	Epigallocatechin gallate	CHIKV	Inhibits the virus attachment		6.54		[190]
	Baicalein Fisetin Quercetagetin	CHIKV			1.891 8.444 13.85		[191]
*Berberidaceae*	Berberine	CHIKV	Inhibits viral protein synthesis and viral replication				[192]
**Natural source**	**Compound**	**Filoviruses**	**Target**	**CC_50_** **(µg/mL)**	**IC_50_ (µg/mL)**	**SI**	**Reference**
	Eugenol	EBOV	Broad spectrum antiviral activity		1.3 μM		[197]
	p-anisaldehyde	EBOV			2.8 μM		
*Aglaia*	Silvestrol	EBOV	Shutdown of viral protein synthesis				[198]

CC_50_: Half maximal cytotoxic concentration; EC_50_-IC5_0_: Half maximal inhibitory concentration; SI: Selectivity index = CC_50_/IC_50_.
